# Erythromycin Estolate Inhibits Zika Virus Infection by Blocking Viral Entry as a Viral Inactivator

**DOI:** 10.3390/v11111064

**Published:** 2019-11-15

**Authors:** Xiaohuan Wang, Shuai Xia, Peng Zou, Lu Lu

**Affiliations:** Key Laboratory of Medical Molecular Virology (MOE/NHC/CAMS), School of Basic Medical Sciences and Shanghai Public Health Clinical Center, Shanghai Medical College, Fudan University, Shanghai 200032, China; 18111010062@fudan.edu.cn (X.W.); 15111010053@fudan.edu.cn (S.X.)

**Keywords:** erythromycin estolate, ZIKV, viral entry inhibitor, inactivator

## Abstract

Recently, Zika virus (ZIKV) has attracted much attention in consideration of its association with severe neurological complications including fetal microcephaly. However, there are currently no prophylactic vaccines or therapeutic drugs approved for clinical treatments of ZIKV infection. To determine the potential anti-ZIKV inhibitors, we screened a library of clinical drugs with good safety profiles. Erythromycin estolate (Ery-Est), one of the macrolide antibiotics, was found to effectively inhibit ZIKV infection in different cell types and significantly protect A129 mice from ZIKV-associated neurological signs and mortality. Through further investigation, Ery-Est was verified to inhibit ZIKV entry by disrupting the integrity of the viral membrane which resulted in the loss of ZIKV infectivity. Furthermore, Ery-Est also showed inhibitory activity against dengue virus (DENV) and yellow fever virus (YFV). Thus, Ery-Est may be a promising drug for patients with ZIKV infection, particularly pregnant women.

## 1. Introduction

Zika virus (ZIKV), a mosquito-borne enveloped RNA virus, is a member of the genus of *Flavivirus* in the *Flaviviridae* family. The *Flaviviridae* family also contains other enveloped viruses, such as dengue virus (DENV), yellow fever virus (YFV), West Nile virus, and Japanese encephalitis virus, which have resulted in epidemics and threatened public health around the world. ZIKV was initially isolated from a sentinel rhesus monkey in Zika forest, Uganda in 1947 [1,2], and had been perceived as a less harmful virus with sporadically infected human cases, which showed mild symptoms, including fever, malaise, rash, and conjunctivitis. However, since 2007, the threat to public health from ZIKV has steadily increased due to outbreaks in varying places, such as Yap Island, French Polynesia, and Micronesia [3,4]. Thus, ZIKV has been recognized as an emerging arbovirus and received attention globally. More recently, unexpected outbreaks of ZIKV infection have occurred in Brazil and rapidly spread from South America to Central-American and Asian countries, involving millions of residents [5]. Meanwhile, the neurological disorders caused by ZIKV have been declared a global public health emergency by the World Health Organization (WHO) [6].

ZIKV was found to be associated with Guillain–Barré syndrome in the epidemics of French Polynesia, and further damage caused by ZIKV, including severe congenital malformation, optic nerve abnormality, and reproductive system injury, have been discovered in recent ZIKV outbreaks [7,8,9,10]. Microcephaly, the most worrying abnormality caused by ZIKV, is a fetal neurodevelopmental defect with resulting physical and learning disabilities, greatly impacting the quality of life of affected children [11]. As the increasing cases of ZIKV sexual transmission and multiple harmful outcomes to fetuses have been reported, women who are pregnant or trying to conceive are now regarded as at especially high risk for ZIKV infection, and thus the need for effective treatments has become ever more pressing [12]. With the increasing recognition of the epidemiology and pathogenicity of ZIKV infection, it is urgent to develop potent therapies. Currently, there are no vaccines or therapeutic drugs approved to treat ZIKV infection, although great progress has been achieved. Many potent inhibitors, which play roles in suppressing the virus–host interaction and membrane fusion, restraining viral replication and translation, or disturbing autophagy, have been explored and identified in vitro and in vivo [13,14,15]. However, the de novo development of a novel inhibitor requires drug design and validation, which takes an extended amount of time. Therefore, repurposing FDA-approved medical and pharmaceutical products may become an alternative choice to provide immediately available drugs for patients. Many already-approved drugs have been discovered to significantly inhibit ZIKV infection, but few can be safely used by pregnant women because most are highly toxic [16]. Thus, the safety profile for pregnant women in the development of ZIKV inhibitors must be considered a priority.

**Figure 1:**
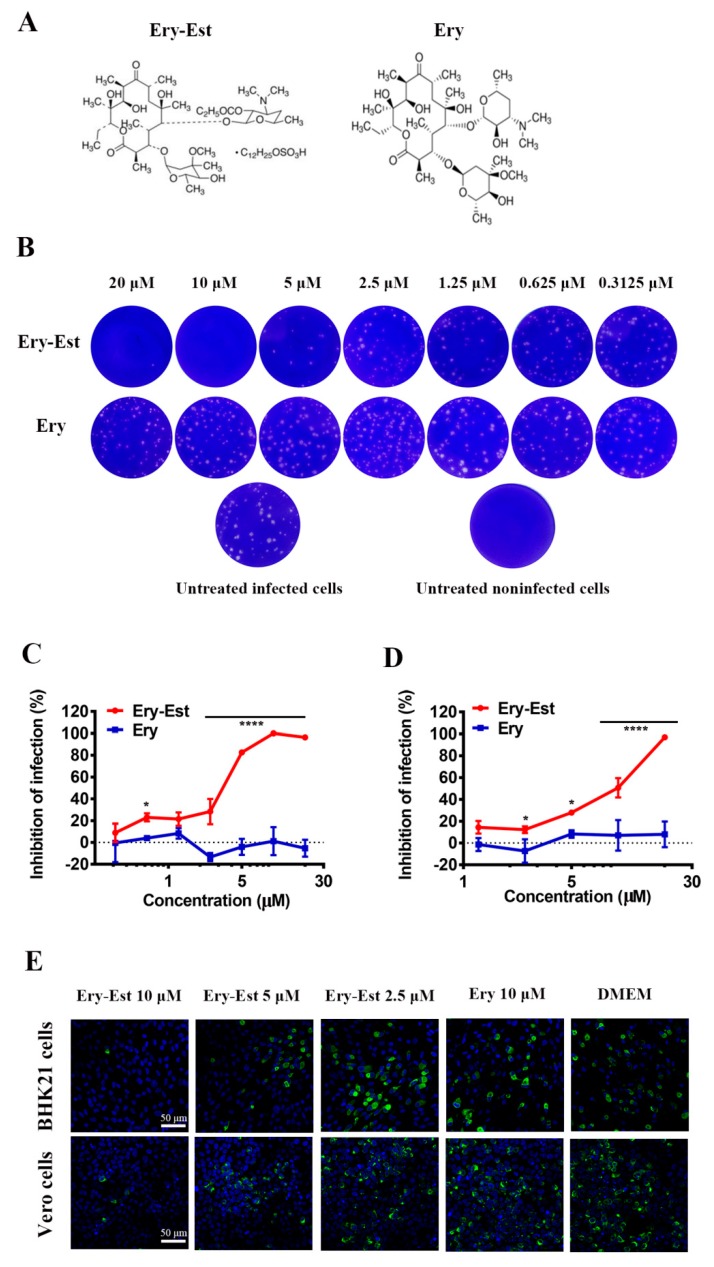
The molecular formula of erythromycin estolate (Ery-Est) and Ery and their inhibitory activity against Zika virus (ZIKV) in different cell types. (**A**) The molecular formula of Ery-Est and Ery. (**B**) BHK21 cells (2 × 10^6^) were infected by ZIKV strain SZ01 that were treated with serial concentrations of Ery-Est and Ery, the infected cells were then covered with an overlay of Dulbecco’s modified Eagle’s medium (DMEM) containing 2% fetal bovine serum (FBS) and 1% low meliting-point (LMP) agarose. After incubation for about 5 days, infected cells were stained with 1% crystal violet for plaque visualization and (**C**) the inhibitions of infection were calculated. The experiment was tested in triplicate and data are represented as means ± SD. (**D**) U-251 MG cells (2 × 10^5^) were infected by ZIKV treated with different concentrations of Ery-Est and Ery. The percentage of infected cells at 48 h post infection was determined by flow cytometry and normalized to untreated infected cells and the inhibitory percentages were calculated. The experiment was tested in triplicate and data are represented as means ± SD. (**E**) BHK21 and Vero cells (2 × 10^5^) were infected with ZIKV strain SZ01 and treated by Ery-Est and Ery, then the ZIKV infection was evaluated by immunofluorescence staining after 4 days. ZIKV E protein were stained by mAb 4G2 (green); nuclei were stained by 4,6-diamidino-2-phenylindole (blue), scale bar: 50 µm. Each experiment was repeated at least twice and similar results were obtained. Statistical analysis: Two-way ANOVA with Sidak’s multiple comparisons for (**C**) and (**D**). * *p* < 0.05; **** *p* < 0.0001.

To determine the ready-to-use inhibitors for ZIKV infection, we screened a drug library consisting of FDA-approved medical drugs with good safety, and erythromycin estolate (Ery-Est), which could be safely used by pregnant women, was found to inhibit ZIKV infection efficiently. Ery-Est, one of the macrolide antibiotics, is the lauryl sulfate ester of propionyl erythromycin (Ery) (Figure 1A), and it has commonly been used for clinical bacterial infections. Besides, some macrolide antibiotics have been studied to treat a wide spectrum of viruses. For example, clarithromycin and the macrolide therapy have been utilized to treat respiratory syncytial virus and Middle East respiratory syndrome coronavirus (MERS-CoV) infection respectively, and ivermectin and azithromycin (Azi) were identified to effectively inhibit ZIKV infection [17,18,19,20,21,22,23]. To the best of our knowledge, this is the first time the anti-ZIKV activity of Ery-Est has been reported. Its antiviral effects in vitro and in vivo were also investigated in the present study. Furthermore, we explored the inhibitory mechanism of Ery-Est against ZIKV.

## 2. Materials and Methods

### 2.1. Cells and Viruses

BHK21, Vero, 293T, Huh7, and U-251 MG cells were cultured in Dulbecco’s modified Eagle’s medium (DMEM, Biological Industries, Israel) with 10% fetal bovine serum (FBS, Biological Industries, Beit HaEmek, Israel) and maintained at 37 °C with 5% CO_2_. The C6/36 cell was grown in DMEM containing 10% FBS at 28 °C with 5% CO_2_.

Three ZIKV strains, SZ01 (GenBank: KU866423), FLR (GenBank: KU820897.5), and MR766 (GenBank: LC002520), were used in this study. SZ01 was originally isolated from a patient who returned from Samoa [24]. Both FLR, isolated from the blood of a human in Colombia [25], and MR766, isolated from a sentinel monkey in Uganda in 1947 [1], were obtained from ATCC. YFV strain 17D was obtained from Beijing Tiantan Biological products, Ltd. and prepared as previously described [26]. DENV II strain New Guinea C was kindly provided by Zhigang Song at Shanghai Public Health Clinical Center. The ZIKV strains SZ01 (passage 6–8), MR766 (passage 4–6) and FLR (passage 4–6), DENV II strain New Guinea C (passage 5–7), and YFV strain 17D (passage 5–7) were used in the experiments. Besides the ZIKV strain SZ01 prepared in Vero cells (V-ZIKV), all flaviviruses used in this study were propagated in C6/36 cells.

### 2.2. Compounds

Erythromycin estolate (Ery-Est), Erythromycin (Ery), and azithromycin (Azi) powders were bought from Santa Cruz Biotechnology, Inc. (Dallas, CA, USA) and MedChemExpress (Shanghai, China), and the lyophilized powder was dissolved in dimethyl sulphoxide (DMSO) and then stored at −20 °C.

### 2.3. Plaque Assay

Viral titers were determined by plaque assay performed on BHK21 cells as previously described [27]. BHK21 cells were seeded in cell plates and incubated to a confluent monolayer at 37 °C with 5% CO_2_ for the experiments. Virus stocks or samples were serially diluted and added to cells, then incubated for 2 h. After incubation, the media of viruses or sample dilutions were removed from BHK21 cells, and the infected cells were covered with an overlay of DMEM containing 2% FBS and 1% low melting-point (LMP) agarose (Promega Co., Madison, WI, USA). The plates with infected cells were further incubated for approximately 5 days, then fixed by 4% formaldehyde and stained with 1% crystal violet for plaque visualization. The titer of virus stock was expressed as plaque forming units (PFU)/mL.

### 2.4. Drug Cytotoxicity Assay

BHK21 and Vero cells were seeded at a density of 2 × 10^4^ cells/well in 96-well plates and incubated at 37 °C overnight. Then, Ery-Est and Ery, serially diluted in DMEM containing 2% FBS, were added to cells and incubated at 37 °C for 3 days. Cell Counting Kit-8 (CCK8, Dojindo, Japan) was used to detect the cell vitality as previously described [28]. In the detection using CCK8, orange-colored formazan could be formed by WST-8 through catalyzing with dehydrogenase in living cells, and the amount of formazan was proportional to the number of living cells. After the absorbance was determined at 450 nm wavelength, the cell proliferation was calculated according to the instruction manual.

### 2.5. Assays for Antiviral Activity

For the drug screen, equal volume of ZIKV strain SZ01 (100 PFU/mL) and compounds diluted in serum-free DMEM at the final concentration of 10 µM were incubated at 37 °C for 1 h. BHK21 cells (2 × 10^4^) were infected with compounds-treated ZIKV at 37 °C for 12 h, followed by replacing the viral supernatant with fresh DMEM containing 2% FBS. When the ZIKV-induced cytopathic effect (CPE) was obvious, CCK8 was used to detect the antiviral activity of compounds according to the instruction manual.

To evaluate the antiviral efficacy against ZIKV (SZ01, FLR, and MR766) and DENV II New Guinea C and YFV 17D, Ery-Est and Ery were serially diluted in serum-free DMEM and mixed with 100 PFU of viruses, and then incubated 1 h at 37 °C. For the plaque assay, the mixture was then added to cells seeded in cell plates. After incubation at 37 °C for 2 h, the supernatant was replaced with DMEM containing 1% LMP agarose and 2% FBS. When the viral plaque became obvious, the plaque visualization was carried out as described above. Besides the plaque assay, CCK8 was also used to test the inhibitory activity. The mixture was added to the cells seeded in 96-well plates and incubated at 37 °C for 12 h, followed by replacing the culture supernatant with fresh DMEM containing 2% FBS. When the ZIKV-induced cytopathic effect (CPE) was obvious, CCK8 was used to detect the antiviral activity according to the instruction manual. Data were collected by microplate reader (Infinite M200PRO, Tecan, Morrisville, NC, USA).

To test the inhibitory activity of Ery-Est against ZIKV in neuronal cells, U-251 MG cells were used. Briefly, Ery-Est and Ery were serially diluted in serum-free DMEM and mixed with 2 × 10^4^ PFU of viruses and then incubated 1 h at 37 °C, and the mixture was then added to U-251 MG cells (2 × 10^5^). After incubation at 37 °C for 48 h, the percentage of infected cells was determined by flow cytometry and normalized to untreated infected cells.

The inhibition of Ery-Est against ZIKV in multiple rounds of infection was performed as previously described [29]. BHK21 cells (2 × 10^5^) were infected by 2 × 10^3^ PFU of ZIKV treated with Ery-Est (20 µM), Ery (20 µM), and vehicle (control) and incubated at 37 °C. The supernatant was collected at different timepoints after infection, and the ZIKV titers of collected supernatants were respectively detected by the plaque assay. Additionally, to test the inhibitory activity of Ery-Est against high viral doses of ZIKV, the experiment was performed with a multiplicity of infection (MOI) of 0.1 as previously described [30]. Briefly, BHK21 cells (2 × 10^5^) were infected with 2 × 10^4^ PFU of viruses treated by Ery-Est (20 µM, 10 µM, 5 µM), Ery (20 µM), and vehicle (control). The supernatant was respectively collected at 48 h post infection, and the ZIKV titers of collected supernatants were detected by the plaque assay.

The pseudotyped MERS-CoV and vesicular stomatitis virus (VSV) were chosen as control enveloped viruses and prepared as previously described [27,31,32]. 293T cells were co-transfected with plasmid encoding VSV-G protein or MERS-CoV S protein and PNL4-3.luc.RE using VigoFect (Vigorous Biotechnology, Beijing, China). After 48 h, the supernatants of pseudotyped MERS-CoV and VSV were collected to infect Huh7 cells. The inhibitory activity of Ery-Est on these viruses was measured after 3 days by the luciferase assay system (Promega Co., Madison, WI, USA).

### 2.6. Immunofluorescence Staining Assay

To confirm the inhibitory activity of Ery-Est on ZIKV infection, BHK21 and Vero cells were seeded onto coverslips at a density of 2 × 10^5^ cells/well in 24-well plates and incubated for 24 h. The mixture of 2 × 10^3^ PFU of ZIKV and the equal volume of serially diluted Ery-Est (20, 10, and 5 µM) and Ery (20 µM) was incubated for 1 h at 37 °C and then added to cells. After 24 h, the culture supernatant was replaced by DMEM with 2% FBS. About 4 days later, cells were fixed by 4% paraformaldehyde (PFA; Sigma Aldrich, St Louis, MO, USA), perforated by 0.2% Triton X-100, blocked with 3% BSA (Amresco, LLC, Solon, OH), and incubated with anti-E mAb 4G2 (10 µg/mL) at 4 °C overnight. After 5 washes, the cells were incubated with FITC-labeled rabbit anti-mouse IgG (1:1000, Dako, Glostrup, Denmark) for 1 h at room temperature. After 5 washes, the coverslips were sealed with Prolong Gold Antifade reagent (Thermo Fisher Scientific, Waltham, MA, USA) and scanned with a Leica SP8 confocal microscope.

### 2.7. Time-of-Addition Assay

To determine at which stage the drug displayed inhibitory efficiency, a time-of-addition assay was developed as previously described [27,33]. BHK21 cells were incubated at 37 °C overnight to be infected with 100 PFU of ZIKV. Ery-Est (20 µM) was added to the infected cells at 0, 1, 2, 4, 8, 10 h post infection. After incubation at 37 °C for 12 h, the supernatant was replaced with DMEM containing 1% LMP agarose and 2% FBS. After incubation for approximately 5 days, infected cells were fixed by 4% formaldehyde and stained with 1% crystal violet for plaque visualization.

For the entry experiment, BHK21 cells were infected with ZIKV in the presence of Ery-Est at 37 °C for 1 h, followed by the washes to remove unbound viruses and drugs. For the attachment assay, cells were infected with ZIKV in the presence of Ery-Est at 4 °C for 1 h, and then were washed to remove unbound viruses and drugs. For the fusion assay, BHK21 cells were firstly infected with ZIKV at 4 °C for 1 h to allow viral attachment. After three washes to remove unbound viruses, Ery-Est was added to cells and incubated at 37 °C for 1 h. Then the drug was removed. In the post-entry assay, after BHK21 cells were infected with ZIKV at 37 °C for 1 h to allow viral entry, the drug was added to cells and incubated at 37 °C for 12 h. Finally, the supernatant was replaced with DMEM containing 1% LMP agarose and 2% FBS. After incubation for approximately 5 days, infected cells were fixed by 4% formaldehyde and stained with 1% crystal violet for plaque visualization. Azi, Ery, and DMSO were also included.

### 2.8. Assay to Detect Inactivated Virions

Inactivated virions were separated and detected as previously described [27,34]. Simply, 100 µL Ery-Est and Ery were serially diluted and mixed with 100 µL viruses (500 PFU). After incubation at 37 °C for 2 h, 50% PEG-8000 (Amersco) and 5 M NaCl were added to the mixture at the final concentration of 10% and 0.67 M respectively, and followed by the incubation on ice for 2 h. Then, the treated viruses were centrifuged for 1 h at 20,200 *g* at 4 °C. The supernatant containing the free drugs was removed, and the pellet containing viral particles was washed by 3% PEG-8000 in PBS containing 10 mg/ mL BSA (Amresco, West Chester, PA, USA). After centrifugation, the viruses in the pellet were resuspended in DMEM and the infectivity of viral particles was detected by plaque assay.

### 2.9. RNase Digestion Assay and RT-qPCR

To detect the genomic RNA released from the ZIKV particles, the RNase digestion assay and RT-qPCR were developed as previously described [27,35]. Briefly, Ery-Est and Ery were incubated with ZIKV (1 × 10^3^ PFU) at 37 °C for 2 h. Then, the released RNA from treated ZIKV was digested by micrococcal nuclease (New England BioLabs, Ipswich, MA, USA) for 1 h at 37 °C, followed by the inactivation of the residual RNase. Then, the undigested genomic RNA inside the unbroken virus was extracted using the EasyPure Viral DNA/RNA Kit (Transgen Biotech, Beijing, China) and detected using TransScript II Green One-Step qRT-PCR SuperMix (Transgen Biotech, Beijing, China) and the Master Cycler Ep Realplex PCR system (Eppendorf, Hamburg, Germany) in accordance with the manufactures’ instructions. The primers used to detect the RNA sequences coding viral E protein were as follows: F1 (5′-TGGAGGCTGAGATGGATGG-3′)/R1 (5′-GAACGCTGCGGTACACAAGGA-3′).

### 2.10. Sucrose Density Gradient Assay

ZIKV (1 × 10^6^ PFU) with 100 µM Ery-Est, Ery, 1% Triton X-100, and 1% DMSO treatments respectively or without treatment were incubated at 37 °C for 2 h. Then, the treated or untreated ZIKV was gently loaded onto the top of the sucrose step gradient (20, 30, 40, 50, 60, and 70%), followed by centrifugation in a swinging bucket rotor (SW41Ti, Beckman Coulter, Brea, CA, USA) using an Optima L-100 XP ultracentrifuge (Beckman Coulter) at 107,170 g for 3 h at 4 °C. Fractions were collected from the top to bottom and detected for E protein by western blot and viral genomic RNA by RT-qPCR. Anti-E mAb 4G2 (10 µg/mL) was used to detect the E protein [27].

### 2.11. Ethics Statement

All animal experiments were carried out on the strict basis of ethical guidelines, Animal Welfare Act, and the references of Accreditation of Laboratory Animal Care and other national regulations and rules relating to animals. All animal operational protocols were approved by the Institutional Laboratory Animal Care and Use Committee (IACUC) and the Laboratory Animal Management Ethics Committee at Shanghai Public Health Clinical Center (2016-A021-01) (Approved on 8 September 2016).

### 2.12. Antiviral Efficiency of Ery-Est in A129 Mice

A129 mice used in this study were bred at the Department of Laboratory Animal Science of Shanghai Public Clinical Center under specific-pathogen-free conditions.

To evaluate the protective efficiency of Ery-Est in vivo, A129 mice were used as previously described [27,36]. Forty-eight 4-week-old A129 mice were randomly assigned into six groups and three groups were infected with ZIKV strain SZ01 intraperitoneally (i.p.) at a dose of 1 × 10^5^ PFU/ mouse. At 1 h post ZIKV challenge, infected mice were i.p. administrated with Ery-Est and Ery at 50 mg/kg of body weight or vehicle control (*n* = 8) once a day for 7 consecutive days, followed by observations of body weight, clinical syndrome, and mortality of mice, and it was deemed to be protected if a mouse survived to 21 days post infection (dpi). The viral RNA loads of sera were measured 2 dpi by RT-qPCR. The grade clinical signs were scored as previously described [37]: 0, healthy; 1, lethargy and inactivity; 2, wasting; 3, limb weakness; 4, hind-limb or fore-limb paralysis and tremors; and 5, moribund or death. Mice administrated with heat-inactivated ZIKV (iZIKV) or only administrated with Ery-Est and Ery at 50 mg/kg of body weight were used as mock controls. The above animal studies were conducted in Biosafety Level 2 facility at Shanghai Public Health Clinical Center with Institutional Biosafety Committee approval.

### 2.13. Antiviral Efficiency of Ery-Est in Pregnant C57BL/6 Mice

Antiviral efficiency of Ery-Est in pregnant mice was performed as previously described [27]. Briefly, twenty pregnant C57BL/6 mice (10–12 weeks old, E12–14) were randomly assigned into four groups (*n* = 5). One group infected with heat-inactivated ZIKV (iZIKV) was used as mock controls, and other three groups were i.p. infected with ZIKV strain SZ01 at a dose of 2 × 10^5^ PFU/mouse. After 1 h, infected mice were i.p. administrated with Ery-Est and Ery at 50 mg/kg of body weight or vehicle. At 1 day post infection, pregnant mice were retro-orbitally bled to measure viraemia by RT-qPCR. Three embryos of each mouse were randomly collected to detect viral RNA loads of placentas and fetal heads from those collected embryos.

### 2.14. Statistical Analysis

All statistical analyses were carried out by GraphPad Prism Software 6.0 (GraphPad Prism Software Inc., CA, USA). The log-rank (Mantel Cox) test was conducted to compare the survival curves. Data was given as mean ± SD as indicated. * *p* < 0.05 was considered significant. “*n*” refers to the sample size.

## 3. Results

### 3.1. Ery-Est Inhibited ZIKV Infection in Different Cell Types

To determine the inhibitory activity of Ery-Est against ZIKV, the plaque assay was used as it could precisely exhibit the inhibitory effects. Ery-Est and Ery of serially diluted concentrations were incubated with ZIKV strain SZ01 at 37 °C for 1 h, and then the treated viruses were added to BHK21 cells, which were reported to be susceptible to ZIKV and develop obvious cytopathic effects (CPE) after infection [38], and the ZIKV-forming plaques were presented visually by crystal violet dyeing. As shown in Figure 1B, numbers of ZIKV plaques were reduced more as the concentration of Ery-Est increased. There were scarcely any plaques formed with the Ery-Est treatment of 10 µM or more, while plaques treated by Ery of any concentrations used in the assay were generally unchanged. This suggests that Ery-Est inhibits ZIKV infection in a dose-dependent manner. The 50% inhibitory concentration (IC_50_) value of Ery-Est against ZIKV was 3.22 ± 0.28 µM in the plaque assay (Figure 1C). To test the inhibitory activity of Ery-Est against ZIKV in neuronal cells whose damage caused by ZIKV was especially serious, which would reduce fetal brain growth, U-251 MG cells were infected by ZIKV with serially diluted Ery-Est and Ery treatments. It was found that Ery-Est significantly reduced infection rates of U-251 MG cells with an IC_50_ value of 7.14 ± 0.96 µM (Figure 1D).

We also developed the immunofluorescence staining assay to verify the anti-ZIKV activity of Ery-Est at three different concentrations, and the results were in accordance with the data from the plaque assay. The expression of ZIKV E protein was almost completely blocked by Ery-Est at the concentration of 10 µM and nearly 50% inhibited at the concentration of 5 µM both in BHK21 and Vero cells (Figure 1E). Furthermore, CPE of ZIKV in BHK21 and Vero cells would make cells progress to necrosis and abscission, and their viabilities, thus, could be detected by CCK8, which could evaluate ZIKV infection indirectly. As a result, Ery-Est was testified to inhibit ZIKV strain SZ01 infection with IC_50_ values of 3.55 ± 0.46 and 3.97 ± 0.09 µM in BHK21 and Vero cells respectively (supplementary Figure S1A,B). Taken together, these data indicate that Ery-Est notably inhibits ZIKV infection in different cell types, including neuronal cells.

In addition, to monitor the inhibitory activity of Ery-Est against viral growth, Ery-Est was tested to inhibit ZIKV infection in a multistep growth curve. It was demonstrated that Ery-Est greatly decreased ZIKV titers (supplementary Figure S2A) in multiple rounds of infection, suggesting that Ery-Est has a strong and lasting inhibitory effect on ZIKV infection. Besides, it was also shown that Ery-Est could exhibit inhibitory activity against ZIKV infection even with relatively higher viral doses (Figure S2B), and viral titers were reduced by treatment of different concentrations of Ery-Est. Treatment of 5 µM Ery-Est decreased viral titers by approximately 80% compared with control group, indicating that Ery-Est remained effective against relatively higher dose infection of ZIKV.

### 3.2. Ery-Est Inhibited ZIKV Strains FLR and MR766, DENV II, and YFV 17D Infections

**Figure 2:**
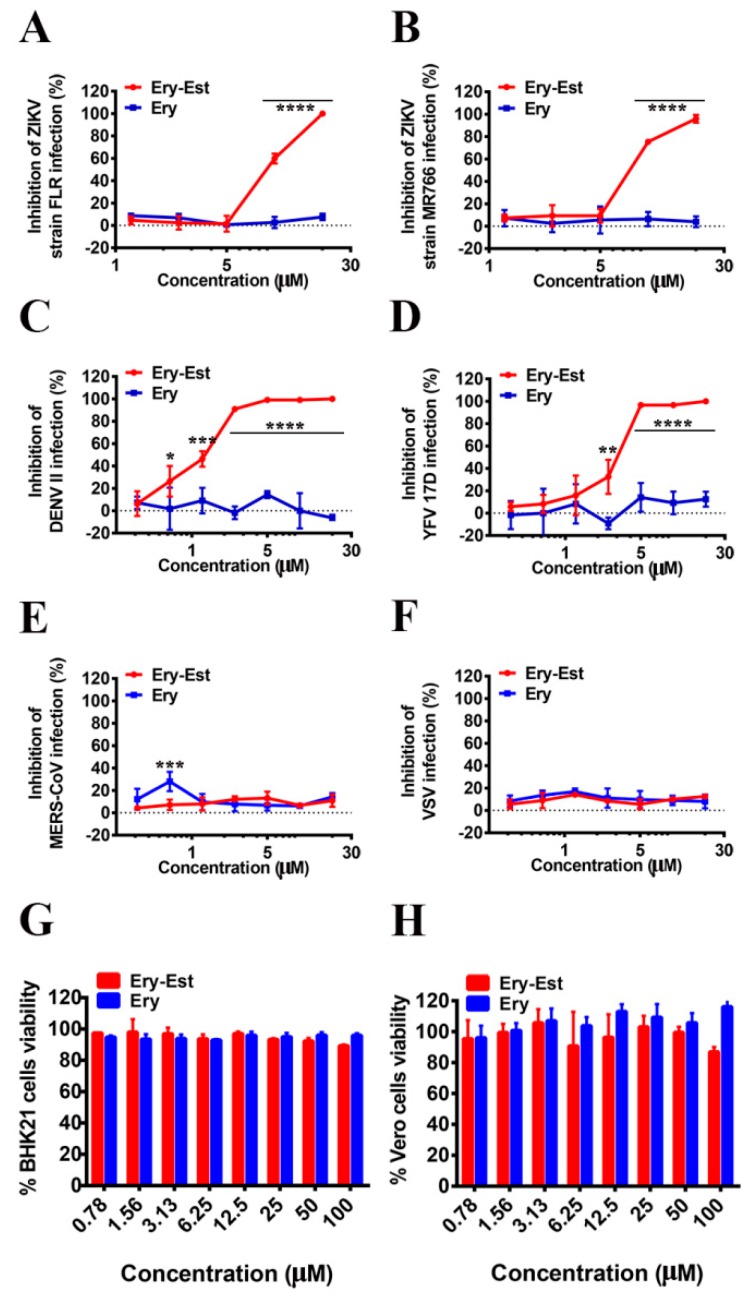
Inhibition of Ery-Est against flavivirus infection with a broad spectrum and its cytotoxicity. BHK21 cells (2 × 10^6^) were infected by ZIKV strains (**A**) FLR, (**B**) MR766, (**C**) DENV II, and (**D**) YFV 17D with the treatment of serial concentrations of Ery-Est and Ery, and then covered with Dulbecco’s modified Eagle’s medium (DMEM) containing 2% FBS and 1% LMP agarose. After incubation for about 5 days, infected cells were stained with 1% crystal violet for plaque visualization and the inhibitions of infection were calculated. Huh7 cells (2 × 10^4^) were infected by (**E**) pseudotyped MERS-CoV and (**F**) VSV with the treatment of Ery-Est and Ery, the inhibitory activity was measured by the luciferase assay systems after incubation for 3 days. (**G**) BHK21 and (**H**) Vero cells (2 × 10^4^) were treated with Ery-Est and Ery for 72 h, and the cell counting kit-8 (CCK8) was used to detect cell viability. The experiments were tested in triplicate and data are represented as means ± SD. Each experiment was repeated at least twice and similar results were obtained. Statistical analysis: Two-way ANOVA with Sidak’s multiple comparisons for (**A**–**F**). * *p* < 0.05; ** *p* < 0.01; *** *p* < 0.001; **** *p* < 0.0001.

As Ery-Est was demonstrated to potently inhibit ZIKV strain SZ01, we then evaluated its inhibitory activity against other ZIKV strains through plaque assay. As shown in Figure 2A,B, Ery-Est significantly inhibited the infections of ZIKV strains FLR and MR766 with IC_50_ values of 7.65 ± 1.77 and 7.15 ± 0.96 µM respectively. Similar to strain SZ01, ZIKV strains MR766 and FLR were approximately half inhibited by micromole Ery-Est, suggesting that Ery-Est could broadly inhibit ZIKV strains that were isolated from rhesus monkeys or patients in different areas of the world.

Following this, we also researched whether Ery-Est was able to inhibit other mosquito-borne flaviviruses, such as DENV and YFV. It was demonstrated that Ery-Est potently inhibited infections of DENV II and YFV 17D with IC_50_ values of about 1.22 ± 0.63 and 2.70 ± 1.03 µM respectively (Figure 2C,D). While Ery-Est, on the other hand, showed no inhibitory activity against other enveloped viruses of pseudotyped MERS-CoV and VSV at the concentration range tested (Figure 2E,F). To rule out the possibility that Ery-Est only inhibits ZIKV prepared in mosquito cells, we further tested whether Ery-Est could effectively inhibit the infection of ZIKV (V-ZIKV) produced in one kind of mammalian cells, Vero cells. Supplementary Figure S3 showed that Ery-Est significantly inhibited V-ZIKV infection with IC_50_ value of about 3.66 ± 0.11 µM, which was similar to the inhibitory activity against ZIKV propagated in mosquito cells. Meanwhile, both Ery-Est and Ery had low toxicity to BHK21 and Vero cells (Figure 2G,H). These results indicate that Ery-Est may effectively inhibit flavivirus infections with a broad spectrum.

### 3.3. Ery-Est Inhibited ZIKV Infection in the Early Stage

**Figure 3:**
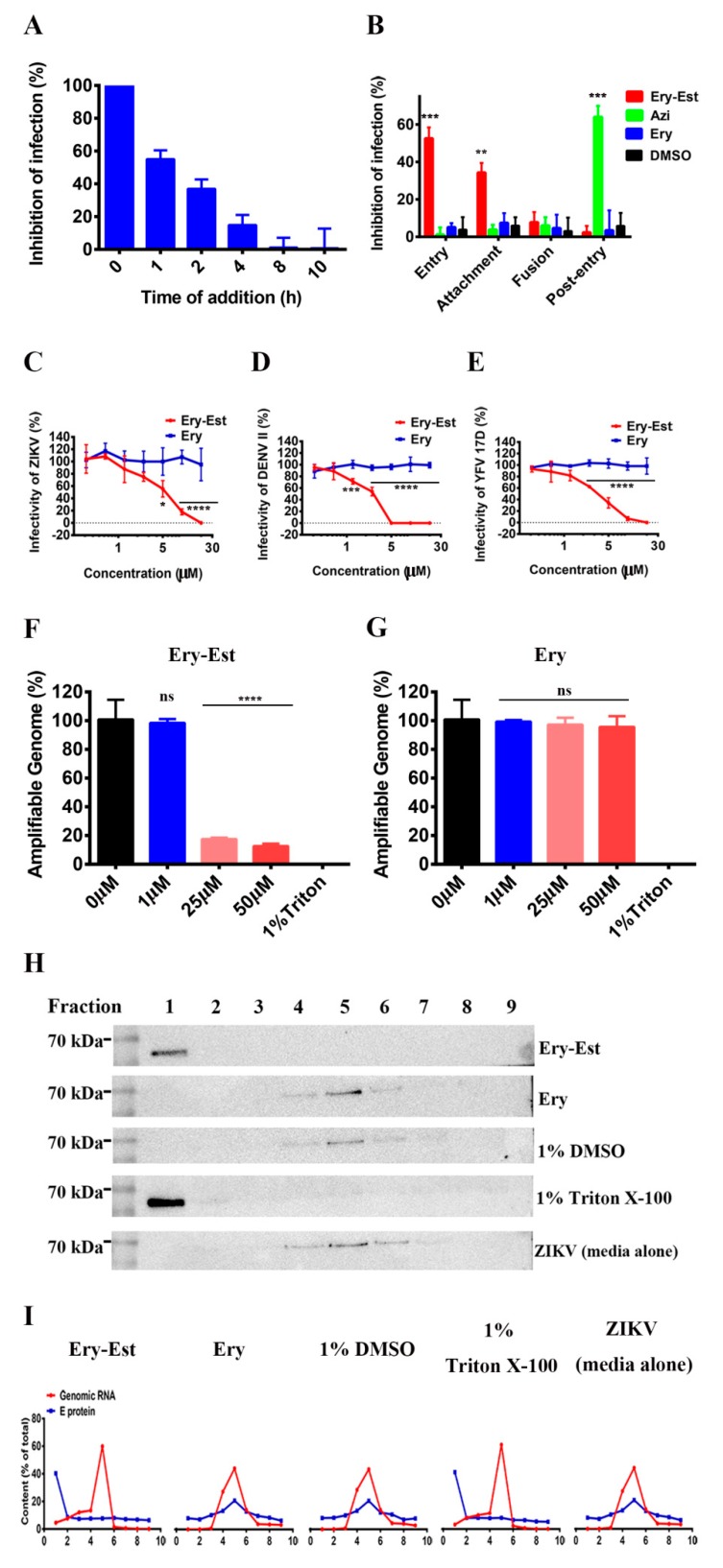
Early stages of ZIKV infection were inhibited by Ery-Est. (**A**) BHK21 cells (2 × 10^6^) were infected with ZIKV SZ01 and Ery-Est was added at different time points post infection. Plaque assay was used to detect viral infection. (**B**) The experimental scheme of viral lifecycle. In the entry experiment, cells were infected with ZIKV SZ01 in the presence of Ery-Est, Azi, Ery, or DMSO at 37 °C for 1 h, and then the unbound viruses and drugs were washed and removed. In the attachment experiment, virus infected cells were presented with Ery-Est, Azi, Ery, or DMSO at 4 °C for 1 h, allowing viral attachment but not membrane fusion due to the low temperature, and washed to remove the unbound viruses and drugs. In the fusion assay, cells were first infected with virus at 4 °C for 1 h for viral attachment and washed to remove the unbound viruses, then incubated in the presence of Ery-Est, Azi, Ery, or DMSO at 37 °C for 1 h to allow virus fusion, and unbound drugs were finally removed. In the post-entry experiment, cells were first infected with virus at 37 °C for 1 h to allow virus entry into cells and washed to remove unbound viruses, then Ery-Est, Azi, Ery, or DMSO was added to infected cells for 12 h. Viral infection of those four experiments were evaluated by plaque assay. (**C**) ZIKV, (**D**) DENV II, and (**E**) YFV 17D were respectively treated by Ery-Est at 37 °C for 2 h, after being separated from Ery-Est by PEG-8000, and the viruses were measured for their infectivity. Degradation of released genomic RNA of ZIKV treated by (**F**) Ery-Est or (**G**) Ery in an RNase digestion assay. The genomic RNA coding E protein was detected. The separation of E protein and of ZIKV treated by Ery-Est, Ery, 1% DMSO, and Triton X-100 or with media alone through a sucrose density gradient assay. E protein in each fraction was assessed by (**H**) Western blot and each percent was analyzed by Image J software and then calculated. (**I**) Genomic RNA in each fraction was separated and each percent of total RNA genome was measured by RT-qPCR. For (**A**) to (**G**), the experiments were tested in triplicate and data are represented as means ± SD. Each experiment was repeated at least twice and similar results were obtained. Statistical analysis: unpaired Student’s t test for (**B**); two-way ANOVA with Sidak’s multiple comparisons for (**C**–**E**); one-way ANOVA with Dunnett’s multiple comparisons for (**F**) and (**G**). ns = not significant. * *p* < 0.05; ** *p* < 0.01; *** *p* < 0.001. **** *p* < 0.0001.

To determine which step of the viral cycle was susceptible to Ery-Est inhibition, we developed the time-of-addition experiment with different time points for Ery-Est addition post ZIKV infection. At 12 h post infection, the supernatant was replaced with maintained medium for plaque assay to evaluate inhibitory effects of Ery-Est at different time points. As shown in Figure 3A, addition of Ery-Est to BHK21 cells at 0 h post infection significantly decreased the plaque numbers caused by ZIKV, showing the potent impact on the reproduction of ZIKV. Ery-Est, with addition time from 1 h to 4 h, showed gradually lower inhibitory activity but barely suppressed virus infection from 8 h post infection. Generally, Ery-Est inhibited ZIKV infection mainly in the early 2 h stage post infection, indicating that Ery-Est inhibits ZIKV infection in the early stage.

Next, to confirm the particular inhibitory periods of Ery-Est against ZIKV, experiments of separate stages that go through viral entry and post-entry stages were carried out as previously described [33]. Azi, belonging to the macrolide antibiotics as well, was used in the experiment to study inhibitory mechanism of different macrolide antibiotics. As shown in Figure 3B, Ery-Est exhibited obvious inhibitory effects on viral entry, but barely inhibited ZIKV infection in the viral post-entry stage. The result was consistent with above time-of-addition assay that showed the inhibitory activity of Ery-Est in the early stage. However, Azi targeted the post-entry stage in the viral life cycle, exhibiting different inhibitory mechanisms from Ery-Est. As viral entry consists of various processes, including viral attachment and fusion, we developed separate experiments to further explore the inhibitory mechanism of Ery-Est. They showed that Ery-Est significantly obstructed the attachment of ZIKV. Overall, these results suggest that Ery-Est may be an entry inhibitor against ZIKV, mainly suppressing the process of attachment, and the inhibitory stage of different macrolide antibiotics may vary widely.

### 3.4. Ery-Est Inactivated ZIKV Virons

The above study of the inhibitory mechanism of Ery-Est demonstrates that it mainly obstructs ZIKV attachment, implying that Ery-Est may bind to viral particles to reduce its infectivity. We, thus, further investigated whether Ery-Est could inactivate ZIKV directly. PEG-8000 was used to separate the treated ZIKV virions from the free Ery-Est after the incubation of ZIKV and Ery-Est or Ery as previously described [34]. The infectivity of treated ZIKV was then detected by plaque assay. Results showed that Ery-Est decreased the infectivity of ZIKV in a dose-dependent manner with 50% effective concentration of 5.21 ± 0.85 µM, while Ery showed no inactivated activity against ZIKV (Figure 3C). Additionally, other mosquito-borne flaviviruses of DENV II and YFV 17D were also used to verify the inactivated effect of Ery-Est. As shown in Figure 3D,E, Ery-Est potently inactivated DENV II and YFV 17D with 50% effective concentrations of 2.56 ± 0.11 and 3.46 ± 0.41 µM respectively. These data indicate that Ery-Est inhibits virus infections through directly inactivating viral particles.

It was reported that some ZIKV inhibitors, such as Z2 and human breast milk, could inactivate virus particles by destructing the integrity of ZIKV [27,39]. Using similar approaches, we investigated whether Ery-Est inactivated ZIKV by inducing the release of genomic RNA from virions. Through the RNase digestion assay, we found genomic RNA from untreated ZIKV (0 µM Ery-Est) or treated by Ery were protected from RNase digestion, but the RNA of ZIKV treated by Ery-Est was digested in a dose-dependence manner (Figure 3F,G), implying the reduction of genomic RNA inside the integrated viral particles. About 80% genomic RNA was digested by the RNase after the treatment of 25 µM Ery-Est, illustrating that Ery-Est may cause the release of genomic RNA to inactivate the ZIKV virions. Next, to further confirm this potential inactivated mechanism, ZIKV with media alone or respectively treated with Ery-Est and Ery, 1% DMSO, and Triton X-100 was centrifuged through the sucrose density gradient as previously described [27,35]. Western blot was used to measure the E protein of ZIKV in each fraction of the above four treated groups, and RT-qPCR was used for genomic RNA detection. Through the percent analysis of each fraction, ZIKV E protein and genomic RNA of Ery, DMSO, and ZIKV (media alone) groups were consistently distributed in the fourth to sixth fractions, but that of Ery-Est and Triton X-100 groups were separated and the E protein was mainly concentrated in the first fraction, while the genomic RNA was almost distributed in the fourth to sixth fractions (Figure 3H,I), implying the destruction of viral integrity. These results indicate that Ery-Est may inhibit ZIKV infection by destructing the viral integrity, resulting in the release of genomic RNA.

### 3.5. Ery-Est Protected A129 Mice from Lethal ZIKV Challenge

**Figure 4:**
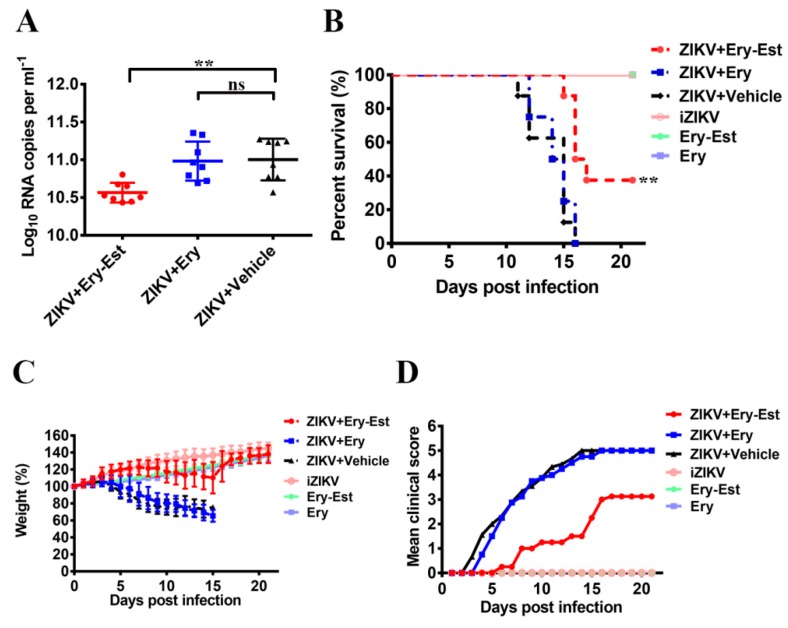
Protective activity of Ery-Est against ZIKV infection in lethal mouse models. Three groups of A129 mice were infected with 1 × 10^5^ plaque forming units (PFU) ZIKV, followed by treatment of Ery-Est, Ery, and vehicle at 50 mg/kg respectively (*n* = 8). Mice administrated with heat-inactivated ZIKV (iZIKV) or only administrated with Ery-Est and Ery were used as mock controls. (**A**) Viral RNA load in sera of A129 mice 2 dpi, mice were retro-orbitally bled to measure the RNA load by RT-qPCR. (**B**) Survival status, (**C**) body weight, and (**D**) clinical score were recorded every day until 21 dpi. Data are presented as means ± SD for (**A**) and (**C**). Statistical analysis: Mann–Whitney test for (**A**); Log-rank (Mantel Cox) for (**B**). ns = not significant. ** *p* < 0.01.

As the results in vitro demonstrated that Ery-Est displayed significant anti-ZIKV activity, the protective efficiency of Ery-Est in vivo was estimated in an established transgenic (type I interferon receptor-deficient) A129 mouse model for ZIKV lethal challenge [40]. Ery-Est and Ery were tested at a concentration of 50 mg/kg once a day for consecutive 7 days. The viral RNA loads of sera were measured 2 dpi by RT-qPCR. As shown in Figure 4A, viremia detected 2 dpi was considerably reduced in the mice with Ery-Est treatment, implying they may present better clinical status. By continuous 21-days observation, we found ZIKV-infected mice treated with Ery and vehicle died within 16 dpi with severe clinical neurological symptoms, including limb weakness, paralysis and tremors. Fortunately, the mice treated with Ery-Est were protected with statistical significance in comparison to vehicle group (Figure 4B), and the surviving mice developed no obvious neurological symptoms. Besides, mice treated with Ery-Est generally kept good weight maintenance and growth (Figure 4C), and they showed lighter clinical signs (Figure 4D) compared to mice with Ery or vehicle treatment. Mice in mock control groups were in good condition. These results suggest that Ery-Est efficiently protects mice from ZIKV-associated neurological signs and mortality. In general, consecutive treatments of Ery-Est showed considerable anti-ZIKV activity, although it played an inhibitory role in the early stage, possibly by inactivating ZIKV virions newly produced from the body and suppressing them to infect more target cells.

### 3.6. Ery-Est Protected Against Vertical Transmission of ZIKV in Pregnant C57BL/6 Mice

**Figure 5:**
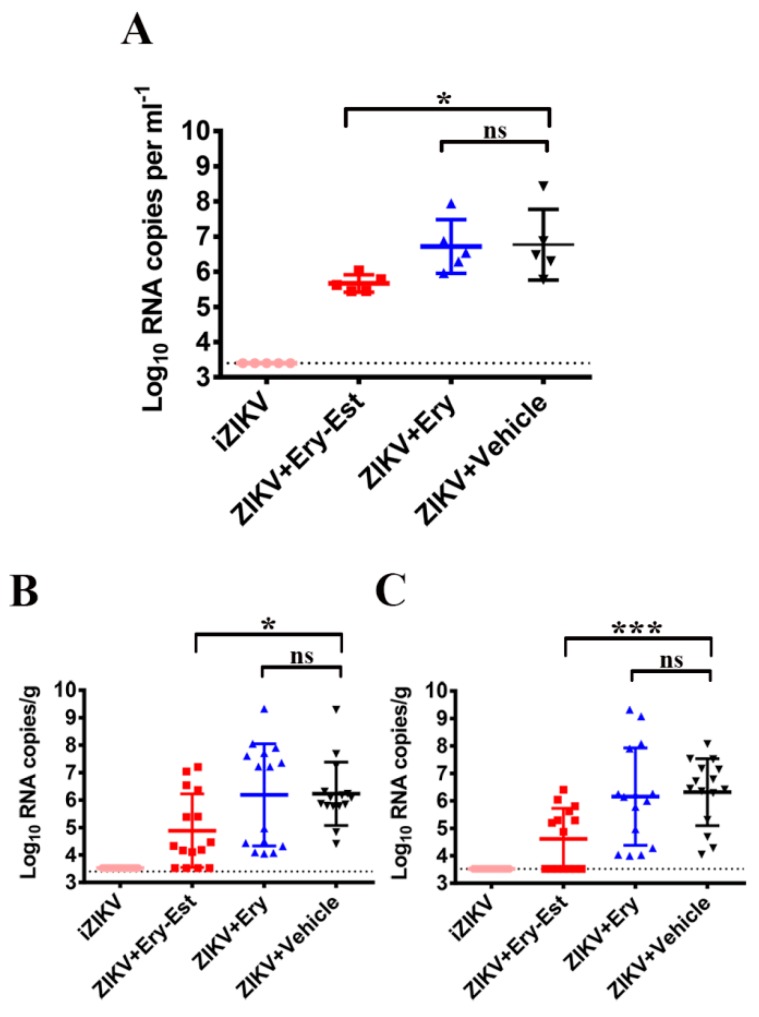
Protective activity of Ery-Est against vertical transmission of ZIKV in pregnant C57BL/6 mice. C57BL/6 pregnant mice were infected by 2 × 10^5^ PFU ZIKV, followed by treatments of Ery-Est (50 mg/kg), Ery (50 mg/kg) and vehicle respectively. Viral RNA loads (**A**) in sera of pregnant mice, (**B**) in placentas, and (**C**) in fetal heads at 1 day post infection were measured by RT-qPCR. Three embryos of each pregnant mouse were randomly collected for placentas and fetal heads. Statistical analysis: Mann–Whitney test for (**A**–**C**). Data are presented as means ± SD. ns = not significant. * *p* < 0.05; *** *p* < 0.001.

To determine whether Ery-Est could block the vertical transmission of ZIKV, pregnant C57BL/6 mice were infected by iZIKV (mock infected controls) or 2 × 10^5^ PFU ZIKV as described previously [27], followed by treatments of Ery-Est (50 mg/kg), Ery (50 mg/kg), and vehicle respectively. It was demonstrated that the viraemia of mice treated with Ery-Est was reduced with statistical significance in comparison to groups of Ery or vehicle treatments (Figure 5A). Meanwhile, the viral load of ZIKV in placentas and fetal heads from pregnant mice with Ery-Est treatment was significantly lower than that from mice with Ery or vehicle treatments (Figure 5B,C). Besides, the infection rates of placentas and fetal heads were decreased by Ery-Est treatment. These results suggest that Ery-Est may directly inactivate some ZIKV virions before they have penetrated to the placenta and then the fetus, thus reducing infection rates of placentas and fetuses, as well as blocking vertical transmission of ZIKV in pregnant mice.

## 4. Discussion

In consideration of worldwide attention to recent ZIKV outbreaks, many efforts have been made to explore potential effective therapies. Currently, there is no approved practicable prophylactic vaccine, and eliminating *Aedes*, the main vector of ZIKV, remains the primary preventive measure. The high throughput screening of already-approved drugs has been carried out to provide anti-ZIKV drug candidates [41,42,43]. By using this method, we found a potent inhibitor, Ery-Est, against ZIKV.

In our study, Ery-Est was demonstrated to inhibit ZIKV infection in a dose-dependent manner in plaque assay (Figure 1B,C and Figure 2A,B). It was also shown that Ery-Est could still suppress viral growth and exhibit inhibitory activity against ZIKV infection even with relatively higher viral dose (Figure S2A,B), and treatment of 5 µM Ery-Est decreased viral titers by approximately 80% (Figure S2B). It was reported that the peak concentration of Ery-Est in plasma was 5.93 ± 2.34 μg/mL (5.61 ± 2.22 µM) after multiple doses [44], indicating that Ery-Est could exert antiviral activity and relieve the viremia in the patient infected with ZIKV.

Ery-Est, one of the macrolide antibiotics, can block the protein synthesis of bacteria and is mainly used for *Staphylococcus aureus* infection and other diverse bacillosis [45]. To the best of our knowledge, the anti-ZIKV effects of Ery-Est had not been previously evaluated at the time of our study. Besides, some macrolide antibiotics, such as azithromycin and ivermectin, have been previously found to effectively inhibit ZIKV infection. Moreover, azithromycin was reported to not only inhibit ZIKV in vitro, but also to prevent ZIKV-induced lethality in the suckling mouse model [19,20,21,22,23]. Interestingly, the inhibitory mechanisms of different macrolide antibiotics varied, such as Ery-Est and Azi in the paper (Figure 3B), probably caused by different effects on viral particles and host cells due to their different structures. Further research about why macrolide antibiotics exhibit different inhibitory mechanisms against ZIKV is needed. As the effective macrolides with diverse characteristics and advantages are constantly discovered [19,20,21,22,23,46], more opportunities for research and clinical treatment can be provided.

The clinical dosage of Ery-Est for adults is 0.75–2 g/day according to the drug instructions, and it was reported to be safely used by pregnant women at the dosage of 1 g/day to assess whether antibiotic treatment could benefit pregnant women with heavy vaginal ureaplasma colonization [47]. Since it is considered safe in pregnant women, Ery-Est could provide a choice for ZIKV-infected gravida who are most likely to suffer serious consequences of abortion, premature birth, or having a baby with microcephaly [48,49,50,51]. Meanwhile, as the sexual transmission of ZIKV was constantly reported [52,53], Ery-Est also showed great potential to be developed into the microbicide with both anti-ZIKV and bactericidal effects, which would not only block the sexual transmission of ZIKV but would be conducive to the healthy vaginal environment. The safety profile of Ery-Est for pregnant women and vaginal use, thus, increases clinical options of the treatment and prevention for ZIKV infection. Moreover, Ery-Est was demonstrated to protect against vertical transmission of ZIKV in pregnant mice, indicating it may have a good effect on decreasing fetal infection risk or reducing fetal symptoms when used for infected pregnant women.

In our study, besides the inhibitory activity against different ZIKV strains, Ery-Est was also demonstrated to potently inhibit other mosquito-borne flaviviruses, including DENV and YFV. This presents an opportunity for clinical applications to patients, who may suffer co-infections by ZIKV and DENV, in epidemic areas where the viral vector, *Aedes aegypti*, is prevalent. Moreover, it was reported that preexisting DENV antibodies may aggravate the ZIKV infection through an antibody-dependent enhancement (ADE) effect [54,55], the phenomenon in which certain antibodies promote the entry of some heterogeneous viruses into host cells [56,57]. Modification of therapeutic antibodies may decrease the risk of ADE, but relatively increase the cost of these antibodies [58]. Ery-Est, with inhibitory activity against a broad spectrum of flavivirus, may be the affordable choice for patients with simultaneous infections without the risk of ADE effects.

At present, many micromolecular inhibitors that target viral binding, endocytosis, and replication against ZIKV have been discovered, but their suppressing activities are only effective when the virus attaches to, enters into, or replicates inside the target cell [13,14,30]. Through the study of inhibitory mechanism in the paper, Ery-Est was demonstrated to directly inactivate flavivirus (ZIKV, DENV, and YFV). We speculate it probably interacts with conserved sequences in envelope proteins of flaviviruses first and then destabilizes envelope proteins and lipids of the viral membrane. As a consequence, the integrity of the viral membrane is disrupted which results in the loss of viral infectivity. The detailed inactivation mechanism of Ery-Est remains to be further studied in the future. Ery-Est, as a viral inactivator like Z2 that is a synthetic peptide derived from ZIKV envelope proteins, makes it possible to effectively inhibit ZIKV before viral entry and is, therefore, beneficial to treat infectors with viremia [27]. The viral inactivator, on the other hand, will promptly work once it contacts the virus, which may directly reduce the viral load into target cells. Likewise, Ery-Est may prevent the influx of virions to fetuses. Accordingly, combinational therapies of Ery-Est and other inhibitors with different inhibitory stages against ZIKV may have synergistic effects of improving protection.

In conclusion, Ery-Est, one of the macrolide antibiotics, exhibits powerful anti-ZIKV effects in vitro and in vivo, and shows excellent safety, indicating it could be further repurposed as a novel antiviral drug to treat ZIKV infection in populations with high risk, particularly pregnant women.

## Supplementary Materials

The following are available online at https://www.mdpi.com/1999-4915/11/11/1064/s1, Figure S1: Inhibition of Ery-Est against ZIKV in BHK21 and Vero cells through CCK8 assay. Figure S2: Inhibition of Ery-Est against ZIKV in multiple rounds of infection; Inhibitory activity of Ery-Est against high dose infection of ZIKV. Figure S3: Inhibition of Ery-Est against ZIKV (V-ZIKV) prepared in Vero cells.
