# Supplementary material for: Erythromycin Estolate Inhibits Zika Virus Infection by Blocking Viral Entry as a Viral Inactivator
Source: Viruses. 2019 Nov 15;11(11):1064. doi: 10.3390/v11111064 (PMC6893414; doi:10.3390/v11111064)
Supplement: Supplementary file 1 [file viruses-11-01064-s001.pdf]

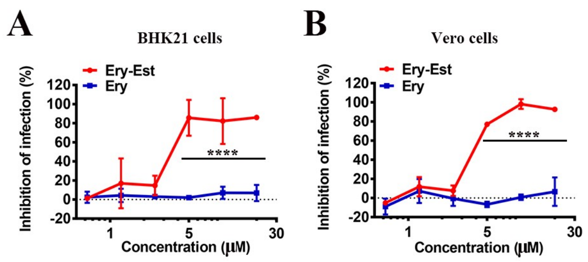

Figure S1. Inhibition of Ery-Est against ZIKV in BHK21 and Vero cells through CCK8 assay. Ery-Est and Ery were serially diluted in serum-free DMEM and mixed with viruses and then incubated 1 h at 37 °C, and the mixture was then added to **(A)** BHK21 and **(B)** Vero cells (2×10^4) and incubated for 12 h, followed by replacing the culture supernatant with fresh DMEM containing 2% FBS. When the ZIKV-induced cytopathic effect (CPE) was obvious, CCK8 was used to detect the antiviral activity. The experiment was tested in triplicate and data are represented as means ± SD. Statistical analysis: Two-way ANOVA with Sidak's multiple comparisons. Data are presented as means ± SD. **** $p < 0.0001$.

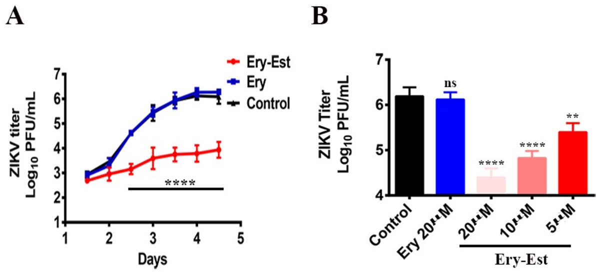

Figure S2. (A) Inhibition of Ery-Est against ZIKV in multiple rounds of infection. BHK21 cells (2×10^5) were infected by ZIKV with Ery-Est and Ery treatment or vehicle (control). ZIKV titers were measured at different timepoints after infection. The experiment was tested in triplicate and data are presented as means ± SD. **(B)** Inhibitory activity of Ery-Est against high dose infection of ZIKV. BHK21 cells (2×10^5) were infected by ZIKV with treatment of Ery-Est, Ery or vehicle (control). ZIKV titers were measured at 48 h post infection. The experiment was tested in triplicate and data are presented as means ± SD. Statistical analysis: Two-way ANOVA with Sidak's multiple comparisons for **(A)**, one-way ANOVA with Dunnett's multiple comparisons for **(B)**. ns = not significant. ** $p < 0.01$; **** $p < 0.0001$.

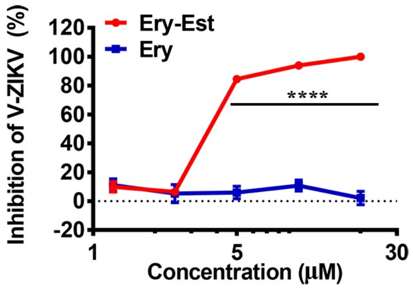

Figure S3. Inhibition of Ery-Est against ZIKV (V-ZIKV) prepared in Vero cells. BHK21 cells (2×10^6) were infected by V-ZIKV that were treated with serially diluted Ery-Est and Ery, the infected cells were then covered with an overlay of DMEM containing 2% FBS and 1% LMP agarose. After incubation for about 5 days, infected cells were stained with 1% crystal violet for plaque visualization and the inhibitions of infection were calculated. The experiment was tested in triplicate and data are presented as means ± SD. Statistical analysis: Two-way ANOVA with Sidak's multiple comparisons. **** $p < 0.0001$.
